# The influence of sex and trauma impact on the rupture site of the ulnar collateral ligament of the thumb

**DOI:** 10.1371/journal.pone.0181754

**Published:** 2017-07-24

**Authors:** Sandra Boesmueller, Wolfgang Huf, Gregor Rettl, Falko Dahm, Alexander Meznik, Gabriela Muschitz, Hugo Kitzinger, Adam Bukaty, Christian Fialka, Martin Vierhapper

**Affiliations:** 1 AUVA Trauma Center Meidling, Vienna, Austria; 2 Medical University of Vienna, Center for Medical Physics and Biomedical Engineering, Vienna, Austria; 3 Medical University of Vienna, Department of Trauma Surgery, Vienna, Austria; 4 Medical University of Vienna, Department of Plastic and Reconstructive Surgery, Vienna, Austria; 5 Medical University of Vienna, Division of General Anaesthesia and Intensive Care Medicine, Vienna, Austria; 6 Sigmund Freud University Vienna, Department of Trauma, Vienna, Austria; University of Pittsburgh, UNITED STATES

## Abstract

**Purpose and hypothesis:**

Although sex- and gender-specific analyses have been gaining more attention during the last years they have rarely been performed in orthopaedic literature. The primary purpose of this study was to investigate whether for injuries of the UCL the specific location of the rupture is influenced by sex. A secondary study question addressed the sex-independent effect of trauma intensity on the rupture site of the UCL.

**Methods:**

This study is a retrospective analysis of all patients with either a proximal or distal bony avulsion or with a mid-substance tear or ligament avulsion of the UCL treated surgically between 1992 and 2015 at two level-I trauma centres. Trauma mechanisms leading to the UCL injury were classified into the following categories: (1) blunt trauma (i.e., strains), (2) low-velocity injuries (e.g., fall from standing height, assaults), and (3) high-velocity injuries (e.g., sports injuries, motor vehicle accidents). After reviewing the surgical records, patients were divided into three groups, depending upon the ligament rupture site: (1) mid-substance tears, (2) proximal ligament or bony avulsions and (3) distal ligament or bony avulsions. Dependencies between the specific rupture site and the explanatory variables (sex, age, and trauma intensity) were evaluated using χ^2^ test and logistic regression analysis.

**Results:**

In total, 1582 patients (1094 males, 488 females) met the inclusion criteria. Mean age was 41 years (range: 9–90 years). Taking into account the effects of sex on trauma intensity (p<0.001) and of trauma intensity on rupture site (p<0.001), mid-substance tears occurred more frequently in women, whereas men were more prone to distal ligament or bony avulsions (p<0.001). In other words, sex and rupture site correlated due to the effects of sex on trauma intensity and of trauma intensity on rupture site, but taking into account those effects there still was a significant effect of sex on rupture site.

**Conclusions:**

The results of this study demonstrate that with regression analysis both sex and trauma intensity allow to predict rupture site in UCL injuries.

## Introduction

Although the importance of sex-specific (referring to the genotype) or gender-specific (referring to the social constructs overlying the genotype) analyses has been pointed out increasingly during the last years [1–4] such investigations are performed in less than one-third of orthopaedic studies [5]. Concerning traumatic ligament injuries, sex differences have not been analysed outside the context of anterior cruciate ligament tears [6–11]. Within this field, a variety of intrinsic factors responsible for ACL tears have been proposed—such as ligamentous laxity [12, 13], the hormonal effects of oestrogen [14] and anthropometric differences [15] between men and women. Other studies have refuted these assumptions [8, 16–18].

Potential sex differences in injuries to the ulnar collateral ligament (UCL) of the thumb have been neglected so far—though they are common with an incidence of 50/100,000 per year. UCL ruptures either occur due to chronic repetitive valgus strain [19] (gamekeeper’s thumb), or to acute excessive valgus stress [20] as seen in skiing accidents (skier’s thumb) and other sports injuries. A special form of this injury is the so-called Stener lesion [21], in which the proximal portion has dislocated over the adductor aponeurosis leading to soft-tissue interposition between the torn ends, thus preventing healing and causing chronic instability [22]. Failure to address laxity of the metacarpophalangeal joint (MCPJ) of the thumb can lead to compromised grip, pain, and ultimately osteoarthritis.

A focused examination of the MCPJ of the thumb should include a methodical assessment of valgus laxity. Instability under valgus stress with the lack of a firm end point is a strong indicator of a complete rupture of the UCL. Valgus stress testing should be performed in both extension and 30 degrees of flexion, so as to determine the status of the accessory and proper collateral ligaments, respectively. The use of stress radiographs has been described for diagnosing UCL injuries—characterized by a laxity of over 35 degrees of abduction in 30 degrees of flexion or extension, or 10 to 15 degrees of increased abduction relative to that of the contralateral thumb [23, 24].

Bony avulsions with a non-rotated, non-displaced bony fragment presenting stable at original assessment can be treated non-operatively in a plaster cast [25]. For instable bony avulsions or complete ruptures in an acute setting, surgical treatment is favoured, as it has yielded excellent results [22, 26]. Bone anchors are currently most commonly used for surgical repair in proximal or distal ligament avulsions, whereas mid-substance tears can also be sutured directly.

A considerable number of studies dealing with UCL injuries can be found in the literature [22, 24, 27–31]–most of them either are surgical reviews [24, 27, 31] or biomechanical studies [28, 29]. Only 2 investigations include larger patient populations [22, 30], and both present demographic data as well as mechanisms of trauma.

However, emphasis has not been placed on potential sex differences in UCL injuries so far. As such, the purpose of this retrospective study was to investigate, by means of demographic analysis, whether sex differences do indeed exist in the framework of thumb UCL injuries—and if so, to determine whether these potential differences have an impact on trauma effects and the variety of UCL ruptures presenting in the clinical setting.

In short, we intended to describe the mechanisms of trauma (i.e., the trauma intensity) leading to UCL injuries of the thumb and present the accompanying relevant demographic data, with a focus on potential sex differences.

## Material and methods

### Patients

This study is a retrospective analysis of all patients with either a proximal or distal bony avulsion or with a mid-substance tear or ligament avulsion of the UCL treated surgically treated between 1992 and 2015 at two level-I trauma centres (which, in combination, are responsible for treating about 50% of the patient population in Vienna, Austria). The study was approved by the Ethics Committee of the Medical University of Vienna and the Vienna General Hospital (approval number: 1467/2015). The need for informed consent was waived since the study was carried out as a retrospective data analysis. All database files and medical records were reviewed for clinical and demographic data (age, sex, trauma history and type of injury); additionally, the description of the UCL rupture site was obtained from surgical records in case of ligament avulsions. In case of bony avulsions the rupture site was identified on plain x-rays.

Inclusion criteria were as follows: (a) patients with a type I (mid-substance tear), type II (proximal or distal ligament avulsion) or type III (proximal or distal bony avulsion) lesion according to Pechlaner et al. [32], (b) patients who had undergone surgery for a type I or type II lesion, (c) patients who presented with a recent injury (i.e., less than 2 weeks from time of trauma) and (c) patients for whom a complete data set was available (i.e. where the location of the ligament avulsion was clearly stated in every operative note). Excluded from this study were patients suffering from partial tears or a dislocation of the MCPJ, patients with lacerations, patients whose UCL had been previously injured or surgically treated, patients with an incomplete data set and patients who presented with non-recent injuries (i.e., later than 2 weeks after trauma).

### Methods

Trauma mechanisms leading to the UCL injury were classified into (1) blunt trauma (i.e., strains, overexpansion leading to UCL rupture), (2) low-velocity injuries (e.g., falls from standing height, assaults) and (3) high-velocity injuries (e.g., sports injuries, motor vehicle accidents).

In both centres the diagnosis for complete UCL rupture was made based on physical exam using stress radiographs showing a laxity of over 35 degrees of abduction in extension.

According to the surgical records, patients were divided into 3 groups depending upon the described rupture site of the ligament: (1) mid-substance tears, (2) proximal ligament or bony avulsions and (3) distal ligament or bony avulsions.

### Statistics

A power analysis indicated that a sample size of about 1448 patients would provide 80% statistical power (at a significance level of 0.05) to detect a difference in the distribution of UCL rupture sites between males and females based on an initial effect size estimate of 0.082 for a two-way probability table [33, 34]. Roughly extrapolating from the number of patients seen in the two level-I trauma centres under consideration, we estimated that we needed to examine all relevant medical records for the period of 1992 to 2015.

The dependent (or response) variable was rupture site (proximal ligament or bony avulsions and mid-substance tears versus distal ligament or bony avulsions). The independent (or explanatory) variables were sex, age, and trauma intensity.

Descriptive statistics (mean ± standard deviation, median, range, and proportions) were calculated for the entire patient cohort as well as for subgroups as appropriate. For inferential statistics, on the one hand χ^2^ test was employed to decide on the existence of a statistically significant dependency between two categorical variables. On the other hand, logistic regression analysis was used to estimate the dependency of a categorical variable and a combination of categorical and continuous variables. We employed Wald test as an overall test for model significance for the latter. Statistical analyses were done using the free software environment R version 3.3.1 (R Core Team, 2013) on a PC running Linux Ubuntu version 16.04.1 LTS.

## Results

A total of 947 patients with a mid-substance tear or a proximal or distal ligament avulsion were treated surgically for a UCL injury of the thumb between 1992 and 2015 at the two level-I trauma centres under consideration. 153 patients, among them 85 males (56%) and 68 females (44%), had to be excluded from final analysis due to the following reasons: non-recent injury (n = 91), dislocation of the MCPJ (n = 33), laceration (n = 6), previously surgically treated (n = 3), or incomplete data set (n = 20). Ultimately, 794 out of 947 patients (427 males and 367 females) met the inclusion criteria and were finally enrolled in this series.

A total of 806 patients with either a proximal or distal bony avulsion of the UCL was found in the databases between 1992 and 2015 at the two level-I trauma centres under consideration. 18 patients, among them 10 males (56%) and 8 females (44%), had to be excluded from final analysis due to the following reasons: non-recent injury (n = 9), explosion injury (n = 2), previously surgically treated (n = 2), or incomplete data set (n = 5). Ultimately, 788 out of 806 patients (667 males and 121 females) met the inclusion criteria and were finally enrolled in this series.

Altogether, 1582 patients were included in this study. Of these patients, 1094 (69%) were male and 488 (31%) were female. Mean age was 41 ± 16 years (range: 9–90 years). The median age for males was 37 years and the median age for females was 48 years.

There was a wide range of causes leading to the UCL ruptures (Table 1): In reference to causality, 143 patients (9%) had suffered a blunt trauma (e.g., strain), 550 patients (35%) had suffered a low-velocity trauma (e.g., trivial fall or assault) and 889 patients (56%) had suffered a high-velocity trauma (e.g., sports injury or motor vehicle accident). Sporting accidents represented the most common mechanism of trauma in our population (n = 804, 51%), followed by trivial falls or assaults (n = 551, 35%). Skiing alone (including snowboarding (n = 20) and cross country skiing (n = 8)) accounted for 35% of injuries (n = 549). 85 patients (5%) suffered a UCL rupture in a motor vehicle accident (MVA) (which includes motorbike accidents, n = 57). Demographics are presented in detail in Table 1.

**Table 1:** Causes of UCL rupture as a function of sex.

Trauma	Subgroups	Male	Female	Total
**Blunt**		116	27	143
**Fall**	Assault (n = 33)	323	227	550
**Skiing**	Skiing (n = 521)	365	156	521
	Snowboarding (n = 20)	14	6	20
	Cross country (n = 8)	3	5	8
**Ball sports**	Soccer (n = 72)	70	2	72
	Basketball (n = 9)	4	5	9
	Volleyball (n = 7)	4	3	7
	Handball (n = 6)	6	0	6
	Tennis (n = 3)	3	0	3
**Biking**		68	24	92
**Others (sports)**		53	13	66
**MVA**	Car (n = 28)	14	14	28
	Motorbike (n = 57)	51	6	57
**Total**		**1094**	**488**	**1582**

There were 75 proximal ligament or bony avulsions (5%), 174 mid-substance tears (11%) and 1333 distal ligament or bony avulsions (84%).

Correlation analyses were performed among the main variables—sex, rupture site and trauma intensity. We found a statistically significant correlation between sex and rupture site (χ^2^ test, p<0.001), showing that mid-substance tears and proximal ligament or bony avulsions occur more frequently in women, whereas men are more prone to distal ligament or bony avulsions (Table 2).

**Table 2:** Distribution of rupture site as a function of sex.

	Proximal (%)	Mid-substance (%)	Distal (%)
**Male (n = 1094)**	41 (3.7)	81 (7.4)	972 (88.8)
**Female (n = 488)**	34 (7.0)	93 (19.0)	361 (74.0)
**Total (n = 1582)**	75 (4.7)	174 (11.0)	1333 (84.3)

Furthermore, there was a statistically significant correlation between sex and trauma intensity (χ^2^ test, p<0.001), showing that men more often tend to suffer UCL injury as the result of a high-velocity trauma. No other sex-specific correlations were found, and we found no significant effect of age on the rupture site.

**Fig 1:**
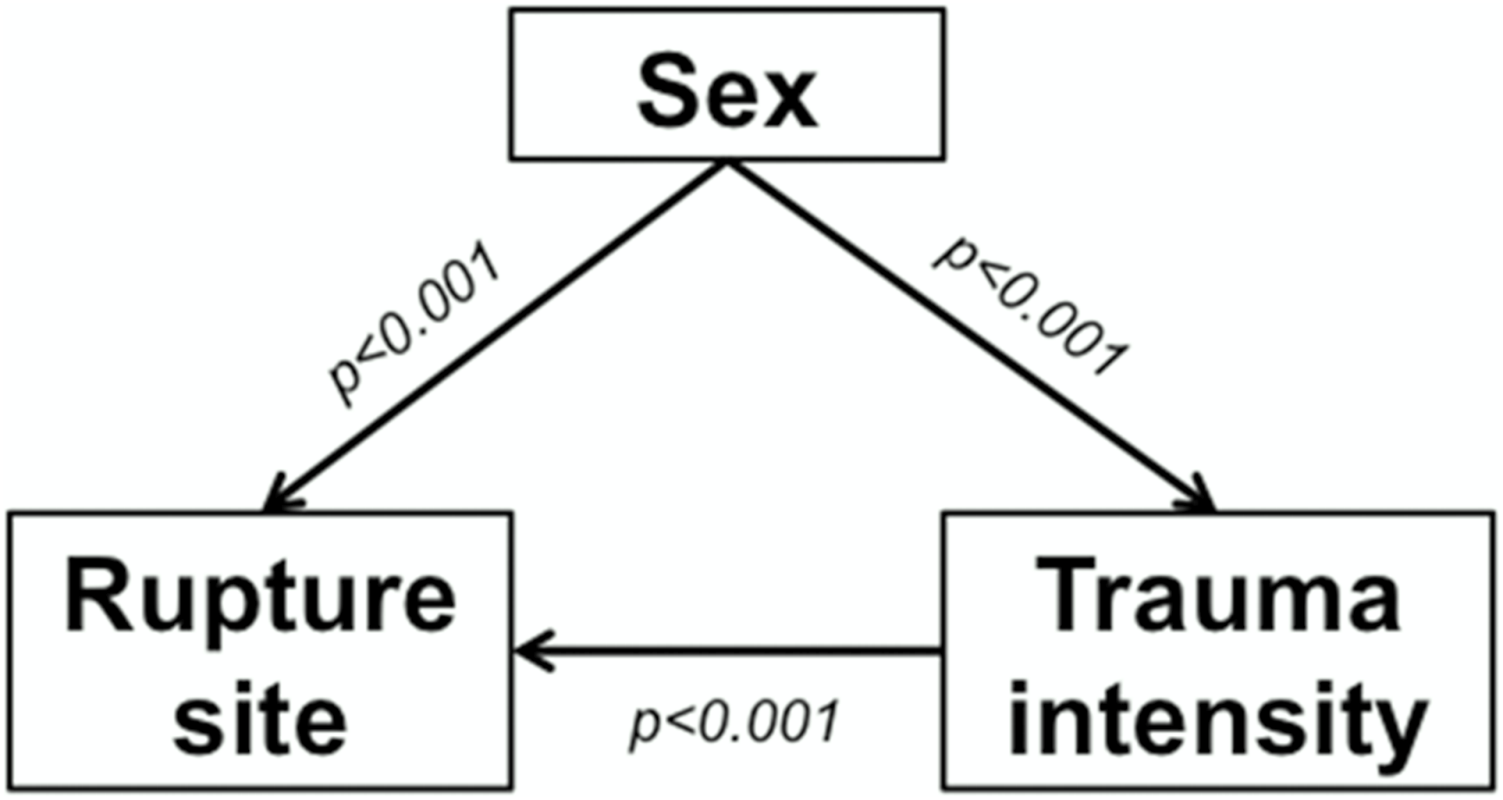
Graphic representation of the study results.

Using a logistic regression model which examined the association of rupture site with sex and trauma intensity, a statistically significant correlation between trauma intensity and rupture site was found—thus showing that a high-velocity trauma results in a distal ligament or bony avulsion in most cases (logistic regression, Wald test, p<0.001) (Table 3). The association between sex and rupture site, however, remained significant even after correcting for the influence of trauma intensity (Wald test, p<0.001). Fig 1 displays a concise graphic representation of the study results.

**Table 3:** Correlation between trauma intensity and rupture site (χ^2^ test, p < 0.001).

	Blunt	Low-velocity	High-velocity
**Proximal (n = 75)**	9	37	29
**Mid-substance (n = 174)**	10	85	79
**Distal (n = 1333)**	124	428	781
**Total (n = 1582)**	143	550	889

## Discussion

The purpose of this study was to expound upon the characteristics of this quite common injury (i.e., UCL rupture) by performing demographic analysis on patient records from two level-I trauma centres—specifically taking into account age, sex, and trauma intensity. Furthermore, we wanted to clinically evaluate if sex differences exist in this context, and whether these potential differences have an impact on the effects of trauma and the variety of UCL ruptures seen in the clinical setting.

In summary, our analysis of 1582 patients represents the largest sample of thumb UCL injuries in the current literature. We were able to show that, in our sample, there exist statistically significant confounder-corrected correlations between sex and rupture site, as well as between trauma intensity and rupture site: Firstly, mid-substance tears occur more frequently in women, whereas men tend to suffer from distal ligament or bony avulsions. Secondly, the higher the trauma impact intensity was, the more distal the UCL ruptures were. As can possibly be expected, men are more prone to high-velocity injuries (i.e., sports injuries or motor vehicle accidents), but this does not fully explain the difference in rupture site between males and females. Rather, there seems to be some sex-specific variable that, by itself, has some meaningful influence on rupture site.

To our knowledge, this is the first study to examine the variety of thumb UCL injuries (proximal or distal ligament or bony avulsions and mid-substance tears) as a function of sex and trauma intensity.

The literature lacks epidemiological data concerning thumb UCL ruptures, as most authors either present surgical reviews [24, 27, 31] or perform biomechanical studies [28, 29]. Moutet et al. [30] published the so far largest study on thumb sprains, identifying the following causes for thumb UCL injuries: skiing, including cross-country skiing (72% of cases); ball sports (13%); other sports injuries (4%). Men were affected in 60% of cases. 62% of patients were under 30 years of age (range: 6–83 years). Although the work of Moutet et al. [30] dates back to 1989, the demographics are very comparable to our study population. However, the authors of that study made no mention of the exact number of verified UCL ruptures.

The probably most interesting study to be compared with ours was performed by Chuter et al. [22], who performed a retrospective review of patients (n = 127) undergoing surgical repair after acute UCL injury. Of those patients, 79 were males (62%) and 48 were females (38%)–yielding a 3:2 ratio. Mean age was 40 years, with a range of 12 to 81 years. The most common causes of injury were falling (49%), sports injuries (19%) and skiing (2.4%). The sex distribution in our study population was comparable to that of Chuter’s [22] as was the age range, though the mean age seen in our study was slightly higher. The most common mechanism of trauma leading to UCL rupture was sports injury (51% of our cases), with skiing accounting for 35% of cases, followed by trivial falls (35%). This observed distribution might be due to the fact that Austria is a well-known, highly frequented skiing destination.

UCL ruptures are known to occur distally in most cases [35, 36]. This is supported by Melone et al. [35], who investigated UCL and radial collateral ligament (RCL) injuries. Unfortunately, the trauma mechanisms were not presented separately for UCL and RCL injuries. However, all UCL disruptions (n = 51) were found to have occurred distally, which might be due to the relatively small study group. Harley et al. [36] performed a biomechanical study in which UCL ruptures were induced in 16 fresh-frozen cadaver thumbs by means of forced abduction and extension. Those researchers were able to show that in 63% of the induced injuries, either the ligament rupture or the bony avulsion took place at the site of UCL insertion. Unfortunately, the authors did not provide any sex-specific information. Our study results show that most ruptures (84%) occur distally at the insertion site. Men suffer more frequently from distal ligament or bony avulsions when compared to women. The higher the velocity involved in the trauma mechanism, the more probable it is that the tear will occur distally. In contrast to injuries of the UCL, Taylor et al. [37] found that the locations of RCL tears were evenly distributed (3 proximal, 4 mid-substance, 4 distal), and that there was no statistically significant effect of sex.

In our study bony and non-bony avulsions were about equally distributed. Interestingly, bony avulsions occurred 5.5 times more often in men than in women which might go against the assumption of bony avulsions being more common in women due to osteopenic bone and thus be an interesting subject for follow-up studies.

As already mentioned, the fact that men are more prone to high-velocity injuries (i.e., sports injuries or motor vehicle accidents) does not fully explain the difference in rupture site between males and females. Rather, there seems to be some sex-specific variable that, by itself, has some meaningful influence on rupture site. Interestingly, high-velocity trauma led to distal ligament or bony avulsions, whereas low-velocity or blunt trauma caused mid-substance tears or proximal UCL avulsions at the ligament’s origin—which might be attributed to an elongating effect of the ligament during low-velocity or blunt trauma.

The major strengths of this study are the large number of patients included, as well as the strict inclusion and exclusion criteria implemented. This study certainly has some limitations with regard to the generalizability of its results—mainly due to its retrospective design. Furthermore, trauma mechanisms and intensities are not always homogeneous, thus, potentially biasing the distinct grouping.

In order to support our findings and to find out if the treatment of UCL injuries of the thumb can be improved or should be changed according to gender disparities, an anatomical study should be performed. Furthermore, a clinical study using a prospective study design is recommended.

## Conclusions

In our study population, the most common mechanisms leading to UCL injury were high-velocity traumas, followed by low-velocity traumas (e.g., falls from standing height). Skiing accidents represented the most common sports-related mechanisms of trauma. Our results indicate a positive correlation between sex and rupture site, as well as sex and the intensity of the trauma leading to the UCL injury. Furthermore, high-velocity injuries lead to distal ligament or bony avulsions in most cases. To our knowledge, this is the first study investigating the impact of sex and trauma intensity on the rupture site of the UCL of the thumb.

## Supporting information

S1 Dataset Patients with a ligament avulsion of the UCL. Patients with a ligament avulsion of the UCL are included in this dataset. To protect the patient’s privacy, the data were aggregated and de-identified from all personal identifiers including (patient ID, birth date, health unit and address). A generic patient id was created to identify unique patients.
(XLSX)

S2 Dataset Patients with a bony avulsion of the UCL. Patients with a bony avulsion of the UCL are included in this dataset. To protect the patient’s privacy, the data were aggregated and de-identified from all personal identifiers including (patient ID, birth date, health unit and address). A generic patient id was created to identify unique patients.
(XLSX)
